# Isotope-reinforced polyunsaturated fatty acids improve Parkinson’s disease-like phenotype in rats overexpressing α-synuclein

**DOI:** 10.1186/s40478-020-01090-6

**Published:** 2020-12-11

**Authors:** M. Flint Beal, Jayandra Chiluwal, Noel Y. Calingasan, Ginger L. Milne, Mikhail S. Shchepinov, Victor Tapias

**Affiliations:** 1 Feil Family Brain and Mind Research Institute, Weill Cornell Medicine, 1300 York Ave, A-501, New York, NY 10065 USA; 2 Deparment of Neurology and Neuroscience, Weill Cornell Medicine, New York, NY 10065 USA; 3 Division of Clinical Pharmacology, Vanderbilt University Medical Center, Nashville, TN 37232 USA; 4 Retrotope, Inc., Los Altos, CA 94022 USA

**Keywords:** Polyunsaturated fatty acids, Lipid peroxidation, α-Synuclein, Gene therapy, Mitochondria, Oxidative stress, Parkinson’s disease

## Abstract

Lipid peroxidation is a key to a portfolio of neurodegenerative diseases and plays a central role in α-synuclein (α-syn) toxicity, mitochondrial dysfunction and neuronal death, all key processes in the pathogenesis of Parkinson’s disease (PD). Polyunsaturated fatty acids (PUFAs) are important constituents of the synaptic and mitochondrial membranes and are often the first molecular targets attacked by reactive oxygen species (ROS). The rate-limiting step of the chain reaction of ROS-initiated PUFAs autoxidation involves hydrogen abstraction at bis-allylic sites, which can be slowed down if hydrogens are replaced with deuteriums. In this study, we show that targeted overexpression of human A53T α-syn using an AAV vector unilaterally in the rat substantia nigra reproduces some of pathological features seen in PD patients. Chronic dietary supplementation with deuterated PUFAs (D-PUFAs), specifically 0.8% D-linoleic and 0.3% H-linolenic, produced significant disease-modifying beneficial effects against α-syn-induced motor deficits, synaptic pathology, oxidative damage, mitochondrial dysfunction, disrupted trafficking along axons, inflammation and DA neuronal loss. These findings support the clinical evaluation of D-PUFAs as a neuroprotective therapy for PD.

## Introduction

Age is the main risk factor for the development of neurodegenerative diseases, including Parkinson’s disease (PD), a severe neurological disorder characterized by the progressive and selective degeneration of dopamine (DA)-producing neurons in the substantia nigra (SN) and axonal projections [31]. A neuropathological hallmark of PD is the presence of Lewy bodies (LBs) in many of the remaining neurons and in the neuropil, which are eosinophilic intracytoplasmic inclusions of aggregated forms of α-synuclein (α-syn). Accumulation of α-syn at the presynaptic terminals alters synaptic function, neurotransmitter release and DA metabolism [22]. α-Syn point mutations, such as A53T and A30P, render the protein more susceptible to aggregation and toxicity and is a cause of familial PD. Viral vector-mediated overexpression of α-syn causes a PD-like phenotype, including gradual nigrostriatal DA degeneration and concomitant motor deficits in rodents and non-human primates [20, 29].

Oxidative damage is a critical process implicated in the gradual loss of neuronal function and in the death of DA neurons. Lipid peroxidation (LPO) has been utilized as an indicator of reactive oxygen species (ROS)-mediated membrane phospholipid degradation, which directly affects cell membrane function. A major deleterious outcome of LPO is the generation of a variety of reactive aldehyde species, such as malondialdehyde (MDA) and 4-hydroxy-2-nonenal (4-HNE). The latter is the most cytotoxic end product and it is detected in neurofilament medium chains and it alters DA uptake, which links LPO to neuronal loss. Postmortem examination of PD brains showed an increased basal content of nigral MDA and the presence of 4-HNE-protein adducts in the SN DA neurons [54]. Elevated levels of cholesterol lipid hydroperoxide were detected by HPLC in the SN of individuals with PD [15]. The concentration of F2-isoprostanes is significantly increased in the anterior cingulate cortex of PD subjects [1].

Polyunsaturated fatty acids (PUFAs) are essential components of the cell membranes and play a pivotal role in brain development and function, epigenetics, and both inflammatory and immune reactions [3]. PUFAs are classified into two main groups, the omega 3 (ω-3) and omega 6 (ω-6) depending on the position of the first double bond. Linoleic and α-linolenic acids are the precursors of long chain PUFAs and they must be obtained through the diet, since mammals cannot synthesize them de novo. PUFAs are prone to LPO, initiated by a single ROS moiety, which results in altered cellular membrane integrity and production of numerous reactive and toxic compounds that induce protein-DNA cross-linking [38]. Recently, α-syn interaction with lipids have been shown to be critical in mediating its toxicity. A lipidomic analysis of α-syn neurotoxicity showed that α-syn triggers release of oleic acid and diglycerides, and that triglycerides and lipid droplets protect against toxicity by sequestering both oleic acid and diglycerides [21]. Preventing lipid droplet formation or augmenting oleic acid levels increases α-syn toxicity. Suppressing the oleic acid generating enzyme stearoyl CoA-desaturase decreased α-syn toxicity in rat neurons, C. elegans models and induced pluripotent stem cells (iPSCs)-derived neurons from PD patients with α-syn triplication. This was accompanied by reversal α-syn inclusion formation. In E46K α-syn transgenic mice, increased levels of lipids followed by progressive motor deficits were found. Unbiased screen studies independently identified stearoyl CoA-desaturase as a modulator of α-syn-mediated toxicity [52]. These observations provide a strong scientific basis for investigating the ability of reducing LPO as a neuroprotective strategy in PD.

It is assumed that an antioxidant therapy may counteract the harmful effects of ROS and therefore, prevent or exert a beneficial effect on oxidative stress-associated diseases. However, reducing the burden of oxidative stress by upregulating the cellular repair processes and/or antioxidant systems is not sufficient in patients with PD. Indeed, some types of damage, such as hydrogen abstraction (C-H bond cleavage) are irreversible, particularly when amplified through a chain reaction format, as in LPO. Our strategy is to stabilize PUFAs by replacing atoms in bonds that tend to be weak spots for ROS attack with a heavier isotope of the same atom, thereby slowing down the rate-limiting step of ROS-driven hydrogen abstraction (Additional file 1: Figure S1). Cellular components that incorporate heavy isotopes, such as ^2^H (D, deuterium), are more resistant to ROS-driven oxidation. PUFAs are avidly taken up as essential nutrients and only a small fraction (~ 20%) needs to be deuterated in membranes to inhibit LPO. Therefore, we examined whether deuteration of bis-allylic sites of PUFAs would mitigate LPO and slow hydroperoxide-mediated toxic cascades that contribute to neurodegeneration in PD. Our study provides compelling evidence that dietary supplementation with D-PUFAs is protective against α-syn-induced neurotoxicity.

## Materials and methods

### Animals

Four-month-old male Lewis rats (Charles River; Wilmington, MA, USA) were maintained under standard conditions of 12 h light/dark cycle, 22 ± 1 °C temperature-controlled room and 50–70% humidity. Animals were given ad libitum access to food and water and were allowed to acclimate to the vivarium conditions for 2 weeks prior experimentation. All procedures were performed with the approval of the Animal Care and Use Committees of Weill Cornell Medicine.

### In vivo gene delivery and treatment protocol

Rats received an intranigral injection of an adeno-associated viral vector (AAV) serotype 5 containing an AAV2 phospholipase (to make the virus more infectious) encoding the human A53T mutant α-syn under the transcriptional regulation of human cytomegalovirus (AAV5.2-CMV-aSynAT-IRES-GFP-SV40pA). The AAV was obtained from Virovek (Hayward, CA, USA). Viral vector suspension in a volume of 2.0 µL was infused at 0.4 µL/minute over 5 min into the SN using a Hamilton syringe with a 30-gauge needle (20° bevel) at a rate of 0.2 μL/min based on the following coordinates: − 5.2 A/P, ± 2.0 M/L, − 8.2 D/V to bregma. Animals received either AAV5.2 carrying control plasmid containing green fluorescence protein (GFP) without the human α-syn gene (AAV-GFP, used at 2.0 × 10^12^ GC/mL concentration) or AAV5.2 expressing both GFP and human α-syn genes (AAV-h*SNCA*, used at 1.68 × 10^13^ GC/mL). Fluorescent images displayed an extensive transduction of SN DA neurons and striatal DA terminals (Additional file 1: Figure S2).

**Fig. 1:**
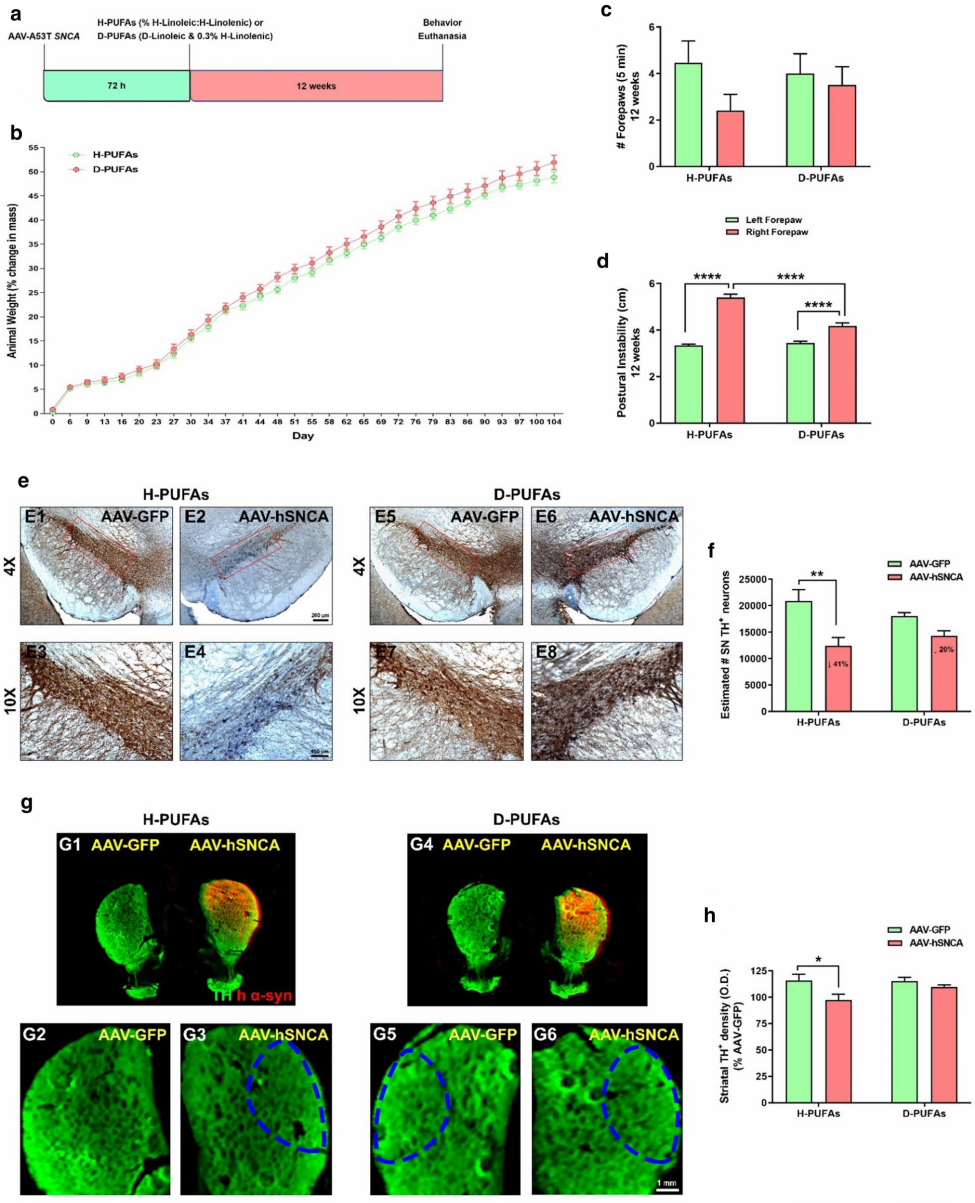
Long-term treatment with D-PUFAs prevents behavioral abnormalities and nigrostriatal DA neurodegeneration in rats overexpressing human α-syn. **a** Experimental design. **b** Percent change in animal body mass. **c** Spontaneous cylinder exploration. **d** Postural instability test. Behavioral assessment was performed at 12 weeks. Values expressed are mean ± SEM of twenty rats per group and analyzed using Mann–Whitney U for rat weight and paired Student’s *t*-test or two-way ANOVA followed by Tukey’s multiple comparisons test for behavior. ****p < 0.0001 and *p < 0.05. **e** Combination of DAB-labeling immunostaining for TH^+^ (brown) with alkaline phosphatase staining for human α-syn (blue) in the rat SN. Representative micrographs were captured at low magnification (4 ×). Insets correspond to high magnification images (10 ×) of the dorsolateral region. **f** Unbiased stereological estimation of the total number of TH^+^-immunopositive neurons. Scale bar: 200 μm for low magnification and 100 μm for high magnification. Nine to eleven SN sections per animal were analyzed. Values expressed are mean ± SEM of six different rats per group and analyzed using two-way ANOVA followed by Tukey’s multiple comparisons test. **p < 0.01. **g** Near-infrared images of the striatum depicting TH^+^ (green) and human α-syn (red) immunoreactive signal. **h** Quantitative analysis of striatal TH^+^ fiber density. Data from 6 sections per animal were pooled and presented as the average mean optical density ± SEM. Experimental groups comprised 5–7 rats. Scale bar: 1 mm. ****p < 0.0001 (two-way ANOVA followed by Tukey’s post-hoc multiple comparisons test)

Thirty-eight rats were randomly divided into two groups and fed 72 h after vector delivery with different dietary amounts of H-PUFAs (control group; 1% H-Linoleic:H-Linolenic) or D-PUFAs (treatment group; 0.8% D-Linoleic & 0.3% H-Linolenic with Blue dye). The isotope-reinforced diet was administered for 12 weeks and was provided by Retrotope (Los Altos, CA, USA). The experimental design is shown in Fig. 1a. The nutritional composition of the chow is shown in Table S1. Animal weight and food intake (~ 18 g/day) were measured two to three times per week. The incorporation of D-PUFAs accounts for ~ 30–40% of total brain PUFAs and this level of D-PUFAs substitution is biologically relevant as approximately 10–20% is sufficient to terminate LPO [42].

### Assessment of functional outcome

To evaluate the deterioration of the nigrostriatal DA system, instinctive exploratory behavior was assessed as described elsewhere [48, 50]. Rats were placed individually into a clear glass cylinder (30 × 20 cm). Experiments were carried out under red-light (10 lx) to encourage movement. Rearing behavior was recorded for 5 min. A rear was defined as the lifting of one or both forelimbs above shoulder level and contacting the wall of the cylinder. The animal must contact the bottom surface with the forelimbs before score another rear. The postural instability test was used to assess forelimb motor function [48, 50]. Rats were held in vertical position facing downward on a tabletop and one forelimb was lightly restrained against the animal’s torso. Next, the body rat was moved forward until making a catch-up step to regain its center of gravity. The new position of the tip of the nose indicated the displacement of the body needed to trigger a catch-up step in the unrestrained supporting forelimb. Tests were carried out at baseline and after 4, 8- and 12-weeks following injection A53T mutant α-syn injection.

### Tissue harvesting and postmortem analyses

Rats were sacrificed under anesthesia (ketamine/xylazine cocktail) and by CO_2_ asphyxiation. Tissue was perfused with 0.9% saline solution to remove the blood. Brains were quickly removed, immersion-fixed in 4% PFA in pH 7.4 PBS for 1 week, and then cryoprotected in 30% sucrose in PBS for a minimum of 3 days until infiltration was complete. Brains were then sectioned in the coronal plane at 35 µm. Free-floating sections were collected in cryoprotectant and maintained at − 20 °C for immunohistochemical analyses. Other rat brains were harvested, and the areas of interest were immediately dissected, snap-frozen in liquid nitrogen and kept at − 80 °C for RT-qPCR, Western blotting and mass spectrometry (MS) analyses.

### Immunohistochemistry

Brains were sectioned in the coronal plane at 35 µm and free-floating sections were collected in cryoprotectant and maintained at − 20 °C. For chromogenic detection, SN sections were washed 6 times in 1X PBS for 10 min each and incubated overnight at 4 °C in a 1% Triton X-100 solution, to increase permeabilization. Next, sections were quenched with 3% H_2_O_2_ for 10 min, rinsed in 3 changes of PBS and incubated in blocking solution consisting of 10% NDS in 0.3% Triton X-100/PBS for 1 h. Unless otherwise specified, all incubations were carried out at RT. To label DA neurons, tissue sections were stained with a primary mouse anti-TH^+^ antibody for 72 h at 4 °C plus 1 h at RT to obtain optimal antibody penetration. Following 3 additional washing steps, brain sections were placed in biotinylated secondary antibody prepared in PBS containing 0.3% Triton X-100 and 1% blocking serum for 1 h. Brain sections were then rinsed, incubated with Avidin–Biotin Complex reagent for 1 h and washed 3 times again. The reaction was developed in an 3,3′-diaminobenzidine substrate for 3–5 min. Sections were allowed to dry overnight, dehydrated in an alcohol gradient for 3 min each and Histoclear for 5 min and coverslipped using Histomount mounting solution.

Brain sections used for immunofluorescence labeling were rinsed in PBS 6 times for 10 min each and blocked with 10% NDS solution for 1 h. Sections were incubated in primary antibodies directed against the protein of interest in the presence of 0.3% Triton X-100 to facilitate antibody access to the epitope for 72 h at 4 °C. Tissue sections were washed 3 times and the staining was revealed with appropriate secondary antibodies for 2 h. After 3 rinses in PBS, sections were mounted onto plus-coated slides and coverslipped using gelvatol mounting media. Primary and secondary antibodies used and working dilutions are detailed in Table S2 and Table S3, respectively.

For near-infrared fluorescence detection, tissue sections were washed 6 times in PBS for 10 min each and incubated in 10% NDS blocking solution for 1 h. Next, sections were stained with primary antibodies for 48 h at 4 °C. After 3 additional washing steps, tissue sections were covered with IRDye secondary antibodies for 2 h. Brain sections were rinsed in PBS and mounted using gelvatol.

### Unbiased neuronal counts

The number of SN TH-immunopositive neurons from one hemisphere was estimated stereologically using an optical fractionator unbiased sampling design equipped with a Zeiss Axioskop 2 plus microscope hard-coupled to a MAC 5000 controller module, a high-sensitivity 3CCD video camera system (MBF Biosciences; Williston, VT, USA) and a Pentium IV PC workstation. An experimenter blinded to the treatment group performed all analyses. An entire nigral series was arbitrarily selected and every sixth section was sampled through the entire SN, which resulted in the analysis of 10–13 sections per animal. The sections were analyzed for counting using a 100 × oil immersion objective and only those cells with a visible nucleus that was clearly TH-immunopositive were counted. The counting frame was 45 × 45 × 13 µm (height × width × dissector height) and the sampling grid was 125 × 125 µm. The average final section thickness was 22.67 µm, making the average guard zone 4.8 µm from the top and bottom of the section. Stereological parameters were optimized based on a pilot study in both control and AAV-injected rats to produce a rigorous estimate of nigral DA neurons. The CE Gunderson (m = 1) values were < 0.1 for all animals.

### Striatal DA terminal density

An IR-tagged secondary antibody combined with image capture on a LI-COR Odyssey scanning system (Lincoln, NE, USA) was used to quantify DA nerve terminal density in the striatum as previously described [48, 50]. Serial TH immunolabeled sections were scanned at a wavelength of 680 and 800 nm at highest resolution (v. 3.0). For densitometric assessment of striatal TH fibers, the dorsolateral region of the striatum was outlined and the average pixel intensity for each section was generated.

### High-resolution confocal laser scanning microscopy

Confocal microscopy analysis was used to assess the transduction efficiency, oxidative damage, mitochondrial dynamic protein levels and neuroinflammatory responses in the striatum and/or SN of rats overexpressing human α-syn. Fluorescent images were acquired on a Zeiss confocal microscope under constant power and pinhole aperture and evaluated using the ZEN blue edition (v 2.6) software package. Fluorescence micrographs were obtained with an HCX PL APO CS40x (NA 1.25) oil-immersion objective lens. For quantification, ROIs were outlined around the neuronal perikarya. Immunofluorescence signal was determined for tyrosine hydroxylase (TH), human α-syn, 4-HNE, 4-hydroxy-2-hexenal (4-HHE), mitofusin 2 (Mfn2), optic atrophy 1 (Opa1), dynamin-related protein 1 (Drp1), inducible nitric oxide synthase (iNOS), 3-nitrotyrosine (3-NT) and ionized calcium-binding adapter molecule 1 (Iba1).

### Western blotting

Brain tissue was homogenized in ice-cold stringent radioimmunoprecipitation assay buffer (1:30, w/v, RIPA, Sigma) containing the protease and phosphatase inhibitors by sonication. Quantification of proteins was performed using the Bradford assay. Lysates were either used immediately or stored at − 80 °C. Equal amounts of protein from each lysate were separated by 4–12% gradient SDS-PAGE gels and electrotransferred onto nitrocellulose membranes, followed by immunoblotting with the primary antibodies diluted in a blocking buffer solution. The membranes were then incubated with respective infrared conjugated secondary antibodies for 2 h at RT and rinsed four times with PBS. Densitometry of positively stained bands was quantified using near-infrared fluorescence detection. The optical density of each protein band was normalized to the internal control, β-actin.

### Gene expression analysis and mtDNA copy number

Brain tissue was processed for DNA extraction according to the Qiagen kit (Valencia, CA, USA). RT-qPCR was performed on a StepOne real-time PCR system (Applied Biosystems, Foster City, CA, USA) using the Luna Universal Probe One-Step RT-qPCR master mix (New England Biolabs; Ipswich, MA, USA) and the Taqman Gene Expression Assay primers (Thermo Fisher Scientific; Waltham, MA, USA). Relative concentrations were calculated based on standard curves and normalized to the expression of the nuclear DNA encoded protein ribosomal subunit 18S RNA. The relative mtDNA copy number was determined using the ratio of the mtDNA encoded subunit cytochrome c oxidase to 18S rRNA. Fold changes in gene expression were quantified using the 2^−ΔΔCt^ method. The following TaqMan rat probes were used (ThermoFisher; Waltham, MA, USA): SIRT1, Rn01428096_m1; SIRT3, Rn01501410_m1; SIRT4, Rn01481485_m1; SIRT5, Rn01450559_m1; cytochrome c oxidase subunit IV, Rn00665001_g1;18S rRNA, Hs99999901_s1.

### Mass spectrometry analysis of isoprostanes and prostaglandins

Brain samples were prepared and analyzed for esterified 8-isoprostanes (8-IsoP) and prostaglandins (PGF2α) using gas chromatography-negative ion chemical ionization MS as described previously [19]. Separations were carried out on a DB-wax column (0.25 mm inner diameter × 15 m length × 0.25-μm film thickness). The oven temperature was programmed from 150 to 300 ºC held for 11 min. Samples were injected with a 1µL injection volume. Analyte peaks were obtained from ion chromatograms. [^2^H_4_]-15-F2t-IsoP was used as internal standard (Cayman Chemical, Ann Arbor, MI USA). The major ion generated in the negative ion chemical ionization (NICI) mass spectrum of the pentafluorobenzyl (PFB) ester, trimethylsilyl ether (TMS) derivative of F2-IsoPs, was the m/z 569 carboxylate anion [M-181 (M-CH_2_C_6_F_5_)]. The major ion generated in the NICI mass spectrum of the PFB ester, TMS ether derivative of F4-NPs, was the corresponding m/z 593 carboxylate anion. The ion generated by the [^2^H_4_]-15-F2t-IsoP internal standard was m/z 573.

### Quantification of microglial phenotype

Confocal fluorescent images from midbrain sections were exported and analyzed using the Fiji software (ImageJ, v1.52). RGB color images were converted into 8-bit grayscale files with pixel values ranging from 0 to 255. After creating a region of interest, background was subtracted, and the average pixel intensity was determined. Values for the number of microglial-positive cells and the area occupied by microglia over the total area were generated.

### Statistics

All statistical analyses were performed using GraphPad Prism software v. 8.0. (La Jolla, CA, USA). The Mann–Whitney U test (Wilcoxon rank sum test) was utilized to assess for significant differences in animal weight. Statistical comparisons between two groups were analyzed using the paired Student’s *t*-test. Multiple comparisons were established by two-way ANOVA analysis followed by Tukey’s post-hoc correction. Data values were expressed as mean ± SEM. A value of *p* < 0.05 was considered to be significant for all tests.

## Results

### Chronic administration of D-PUFAs improves α-syn-induced progressive behavioral deficits and DA neuronal death

Viral vector-mediated delivery of α-syn leads to motor deficits and robust nigrostriatal DA pathology in primates and rats [20, 29]. We therefore investigated the potential neuroprotective properties of dietary D-PUFAs supplementation against AAV-mediated α-syn overexpression. In the first set of experiments, we examined whether D-PUFAs could ameliorate an α-syn-associated motor phenotype resembling some aspects of PD (Fig. 1). Stereotactic injection of α-syn did not cause any effect on either postoperative animal weight (Fig. 1b) or survival (data not shown). The cylinder test was utilized to evaluate spontaneous forelimb use and forepaw contacts during exploration of a behavioral arena [48, 50]. Although not statistically significant, spontaneous exploratory behavior was reduced from the onset of the α-syn injections in rats fed H-PUFAs chow, especially at 12 weeks (Fig. 1c). The postural instability test assesses evoked forepaw movements in a postural control paradigm [48, 50]. The displacement of the rat necessary to provoke a compensatory forelimb movement was significantly increased on the α-syn-exposed side relative to the GFP-injected side of rats treated with H-PUFAs (Fig. 1d). However, α-syn-induced motor deficits were attenuated in rats fed with a diet enriched in D-PUFAs, with an increase in spontaneous rearing activity and a decrease in postural instability.

To further examine the effects that D-PUFAs can elicit on the integrity of the nigrostriatal DA system, coronal sections of both SN and striatum were immunolabeled with both TH and human α-syn antibodies and were subjected to unbiased stereology and infrared detection analyses, respectively. AAV5.2-driven overexpression of human mutated A53T α-syn into the rat SN over a period of 3 months produced a significant loss in the number of SN DA neurons (Fig. 1e, f) and striatal DA fiber density (Fig. 1g, h) as compared to the contralateral control side. However, administration of D-PUFAs resulted in the preservation of TH^+^ neurons and their nerve terminal network in the AAV-h*SNCA* transduced hemisphere at the experimental endpoint as compared to the AAV-GFP side.

### Treatment with D-PUFAs diminishes α-syn levels and restores α-syn-associated synaptic pathology

**Fig. 2:**
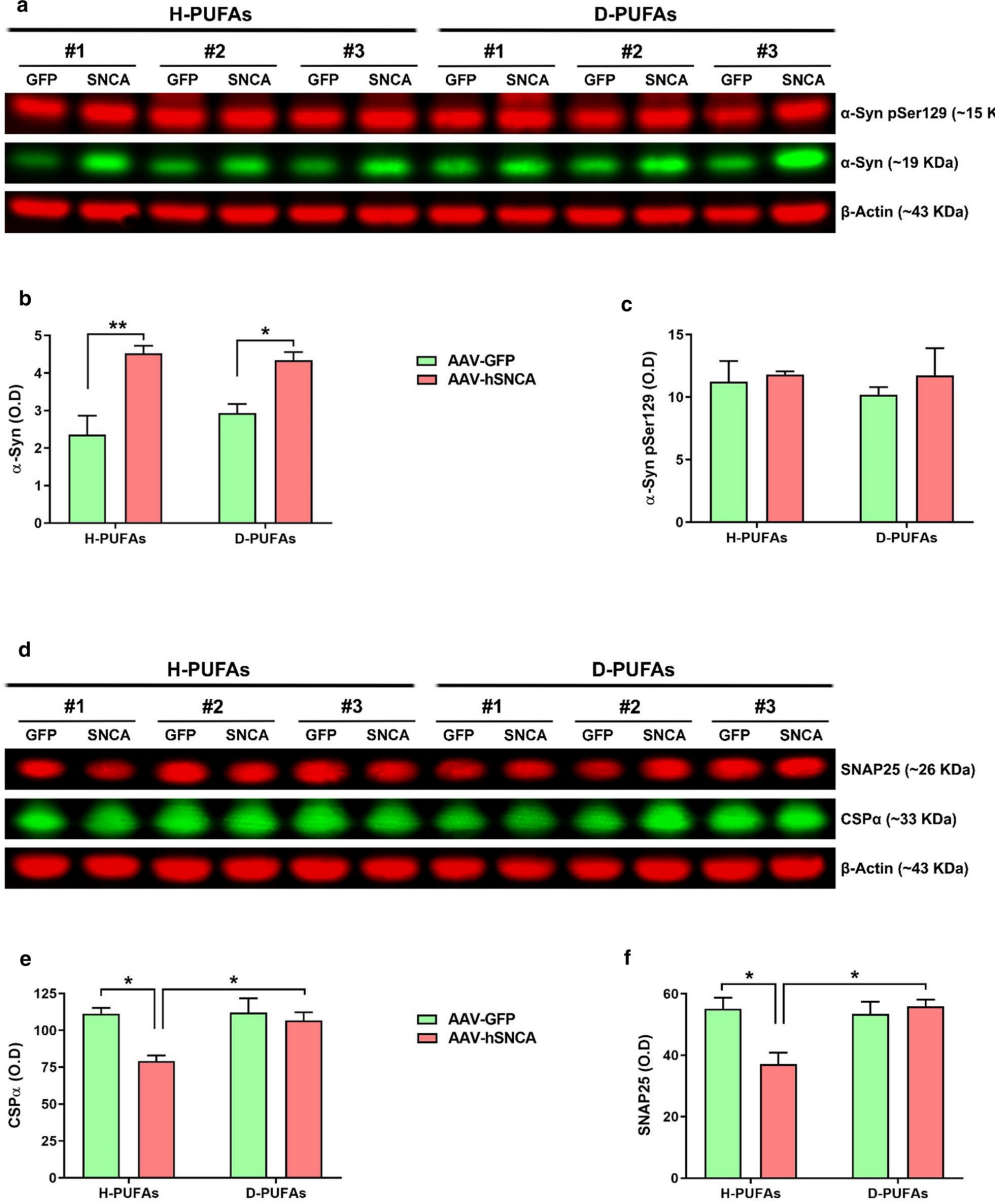
Supplementation with D-PUFAs decreases α-syn content and restores the levels of synaptic proteins. **a** Representative western blot probed with the anti-α-syn and anti-α-syn pSer129 antibodies. **b**, **c** Quantitative analysis of α-syn and α-syn pSer129 protein levels. **d** Immunoblotting detection for the CSP-α and SNAP25 synaptic proteins. **e**, **f** Histogram depicting CSP-α and SNAP25 immunoreactivity. Data were normalized to the loading control β-Actin. Results are expressed as mean ± SEM. n = 3. **p < 0.01 and *p < 0.05. Two-way ANOVA followed by Tukey’s post-hoc multiple comparisons test

α-Syn is predominantly located at the presynaptic terminals where it binds to synaptic vesicles and regulates their trafficking and release. α-Syn accumulation and aggregation can affect synaptic function, including DA release and recycling [22]. The levels of α-syn and synaptic proteins were assessed in this work (Fig. 2). Western blot analysis showed a significant increase in endogenous α-syn immunoreactivity in the AAV-h*SNCA* side as compared to the AAV-GFP hemisphere in H-PUFAs diet-fed rats. The content of monomeric α-syn was significantly abrogated in the α-syn transduced side of rats treated with D-PUFAs (Fig. 2a, b). No changes were observed in the α-syn pSer129 profile (Fig. 2a, c).

Accumulation of pathogenic species of α-syn triggers the onset of synaptic dysfunction in subjects with PD, patient-derived iPSCs and experimental models of PD [10, 49]. We therefore investigated whether D-PUFAs can modulate the content of some synaptic proteins in rat midbrain homogenates (Fig. 2d). Overexpression of α-syn produces a significant reduction in the levels of cysteine string protein alpha (CSP-α; Fig. 2e) and synaptosomal-associated protein 25 (SNAP25; Fig. 2f) compared with the contralateral side of the injection in standard-fed rats. Long-term administration of D-PUFAs returned the synaptic protein content to the basal levels observed in the α-syn injected hemisphere.

### Dietary administration of D-PUFAs mitigates LPO and systemic oxidative damage induced by α-syn

**Fig. 3:**
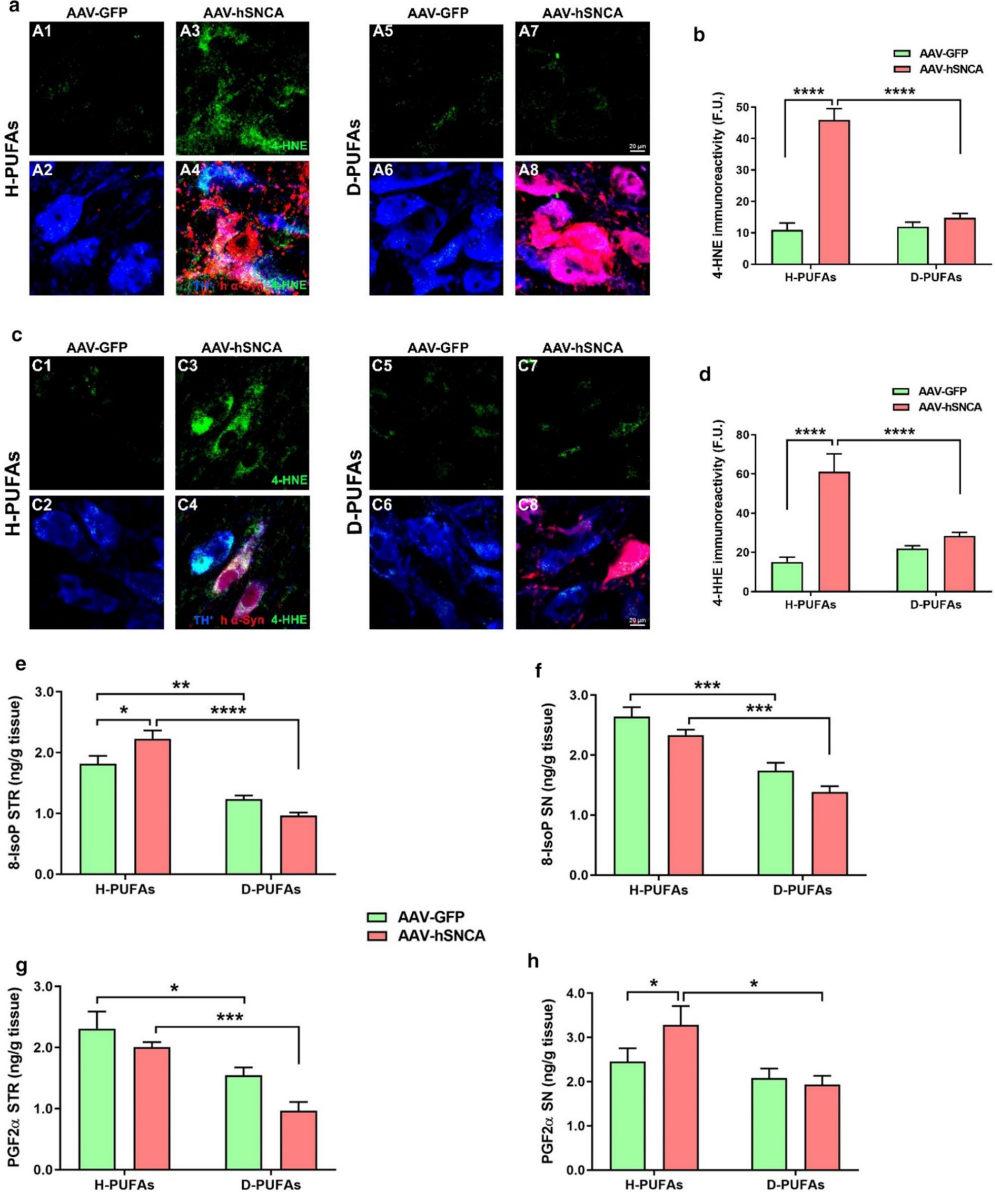
LPO and associated oxidative damage are reduced following administration of D-PUFAs. **a** and **c** Series of high-resolution confocal microscopy images obtained at 40 × of SN sections stained for TH^+^ (blue), human α-syn (red) and 4-HNE or 4-HHE (green). Quantitative assessment of the fluorescence signal of 4-HNE (**b**) and 4-HHE (**d**) in the contralateral and ipsilateral rat SN. Values are mean ± SEM of three sections (average of ~ 150 TH^+^ neurons) per animal. Each group comprised four rats. Scale bar: 20 μm. Content of 8-IsoP in the rat striatum (**e**) and SN (**f**). Levels of PGF2α in the rat striatum (**g**) and SN (**h**). Each value represents the mean ± SEM of seven animals per group. ****p < 0.0001, ***p < 0.001, **p < 0.01 and *p < 0.05. Two-way ANOVA followed by Tukey’s post-hoc multiple comparisons test

**Fig. 4:**
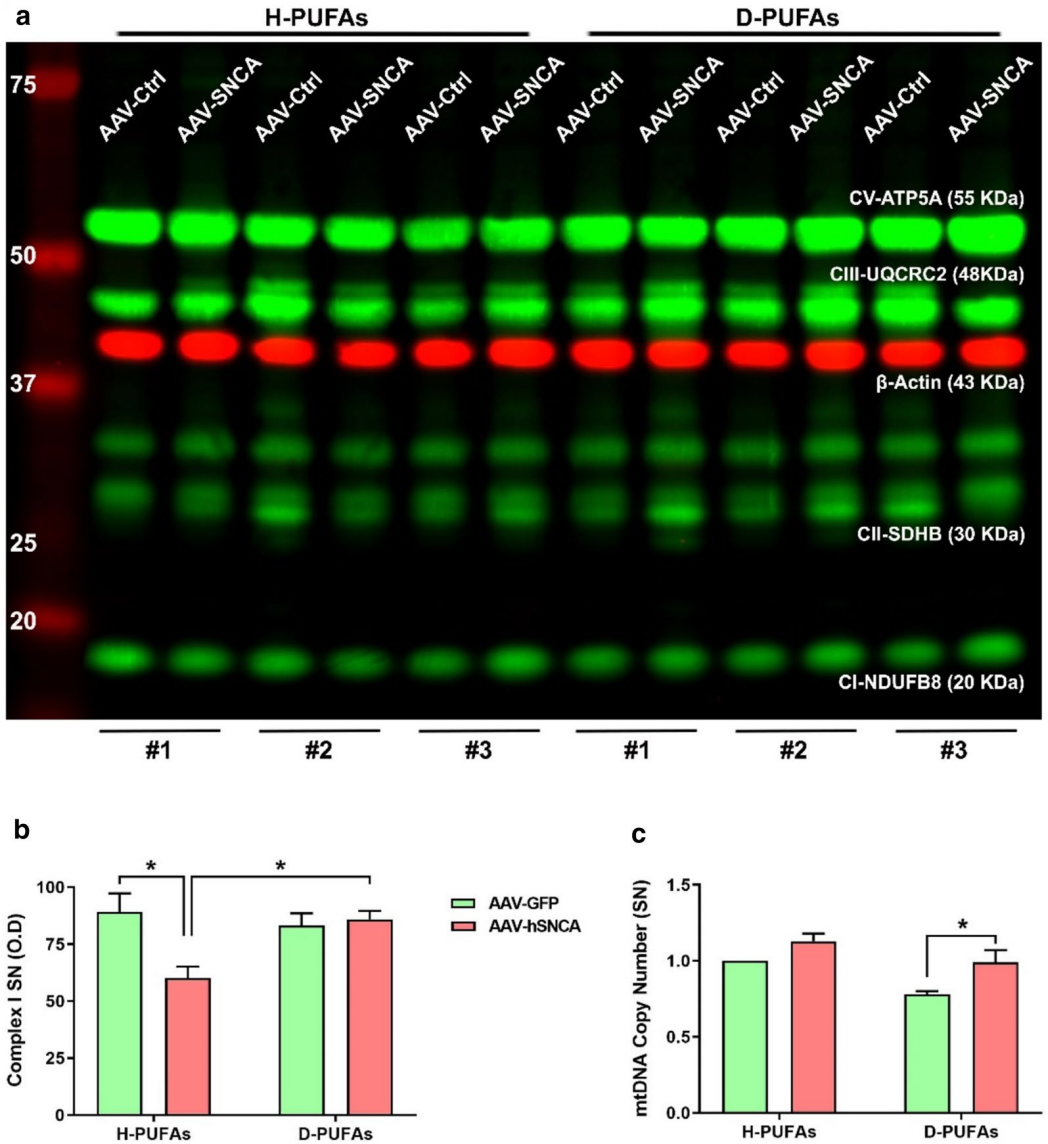
Treatment with D-PUFAs restores mitochondrial function in AAV-A53T α-syn-transduced rats. **a** Western blot for respiratory chain complexes in the rat SN. D-PUFAs preserve complex I immunoreactivity in the ipsilateral hemisphere. **b** Densitometric analysis of each band was used to calculate the relative abundance of the target proteins. **c** mtDNA copy number was determined by RT-qPCR analysis. Bar graphs depict mean values ± SEM of three animals per group analyzed using two-way ANOVA followed by Tukey’s post-hoc multiple comparisons test. *p < 0.05

DA neurodegeneration is caused in part by the ROS-inflicted damage to vital biomolecules. 4-HNE and 4-HHE are specific markers for LPO. A six-fold increase in 4-HNE-positive DA neurons was described in the brains of individuals diagnosed with PD as compared with age-matched controls [54]. We examined whether exposure to D-PUFAs may counteract LPO and the burden of ROS production following α-syn exposure (Fig. 3). Fluorescent images displayed a robust increase in the levels of 4-HNE (Fig. 3a, b) and 4-HHE (Fig. 3c, d) in SN DA neurons of AAV-hA53T α-syn transduced rats fed on a standard chow while the immunoreactivity of those markers returned to control values in animals fed a diet supplemented with D-PUFAs.

Isoprostanes (IsoP) are prostaglandin (PG)-like compounds generated from the free radical-catalyzed peroxidation of essential fatty acids (mainly arachidonic acid). IsoP side chains are predominantly *cis* to the cyclopentane ring whereas PG side chains are in the trans configuration. Both 8-IsoP and PGF2α are recognized as reliable markers of oxidative stress. MS analysis indicated that, at 12 weeks post-injection, H-PUFAs-fed rats had higher levels of 8-IsoP in the striatum of the AAV-h*SNCA* transduced hemisphere in comparison with the contralateral side, although no changes were detected in the SN (Fig. 3e, f). In contrast, the concentration of PGF2α was upregulated in the SN of rats injected with α-syn relative to the GFP-infused side, while no differences were seen in the striatum (Fig. 3g, h). The content of 8-IsoP and PGF2α were downregulated in both hemispheres after administration of D-PUFAs.

### Dietary D-PUFAs improve mitochondrial function in α-syn-injected rats

It has become increasingly evident that mitochondrial dysfunction plays a key role in the pathogenesis of PD. α-Syn has a non-canonical mitochondrial targeting sequence; nevertheless, α-syn can translocate to mitochondria via its N-terminus domain. α-Syn exhibited preferential enrichment in mitochondria from human DA neuronal cultures and the SN of individuals with PD [13]. Herein, we sought to examine the effects of D-PUFAs against α-syn-induced mitochondrial impairment (Fig. 4). In our initial set of experiments, we found that AAV α-syn causes a 30% decrease in complex I immunoreactivity in rat midbrain homogenates (Fig. 4a, b) while administration of D-PUFAs restored complex I levels. We found no changes in striatal complex I protein content (Additional file 1: Figure S3A and B). An instability of the mitochondrial genome has been documented in the brainstem of PD subjects. α-Syn accumulation increased the load of mtDNA deletions in the brains of transgenic mice overexpressing α-syn [5]. Our results showed a significant increase in the mtDNA copy number in the α-syn-transduced SN (Fig. 4c) but not in the striatum (Additional file 1: Figure S3C) of D-PUFAs-fed rats.

**Fig. 5:**
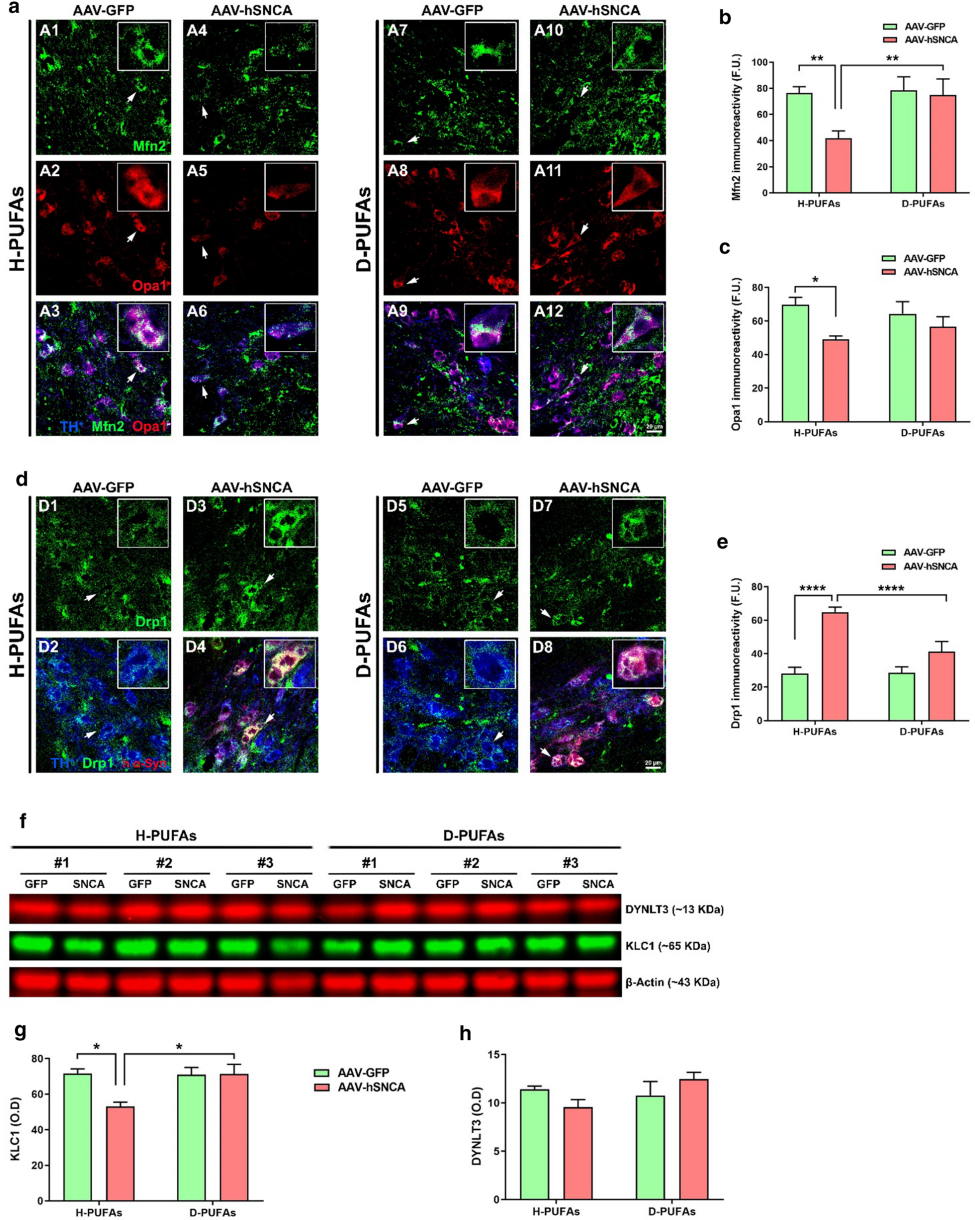
Dietary supplementation with D-PUFAs reestablishes the levels of mitochondrial dynamic- and axonal transport-related proteins in rats overexpressing α-syn. **a** Confocal microscopy images of midbrain sections acquired at 40 × and processed for colocalization analysis between TH^+^ (blue), Mfn2 (green) and Opa1 (red). **b** Mfn2 immunofluorescence signal. **c** Opa1 immunoreactivity. **d** Fluorescence micrographs of SN sections obtained at 40 × and labeled using antibodies against TH^+^ (blue), Drp1 (green) and human α-syn (red). **e** Protein expression profile of Drp1. Scale bar: 20 μm. Each value represents the mean ± SEM of three sections per animal. Four animals per group. **f** Representative blots of fractions stained with KLC-1 and DYNLT3 antibodies. Quantitative analysis of KLC-1 (**g**) and DYNLT3 (**h**) protein levels. Data are expressed as mean ± SEM and represented three rats per group. ****p < 0.0001, **p < 0.01 and *p < 0.05. Two-way ANOVA followed by Tukey’s post-hoc multiple comparisons test

Subtle alterations in mitochondrial dynamics may represent a slow but steady feature disturbing mitochondrial homeostasis. It has been suggested that α-syn is required for the maintenance of mitochondrial integrity by regulating fusion/fission events, transport and clearance [37]. We investigated whether D-PUFAs have an impact on mitochondrial dynamic-related proteins in animals overexpressing human α-syn (Fig. 5). Confocal microscopy analysis of midbrain sections of rats fed with a standard diet demonstrated that, at 12 weeks post-transduction, α-syn intoxication caused a significant decrease in the immunofluorescence signal of the mitochondrial mitofusin 2 (Mfn2) and optic atrophy 1 (Opa1) fusion proteins (Fig. 5a–c) and an increase in the levels of the fission protein dynamin-related protein 1 (Drp1) (Fig. 5d, e) in comparison to the GFP-infused side. Treatment with D-PUFAs abrogated α-syn-related alterations in mitochondrial dynamic protein expression profiles.

### Supplementation with D-PUFAs preserves the levels of axonal transport-associated proteins

Axonal transport, a physiological process critical to neuronal viability and function, is tightly regulated by the kinesin and dynein motor proteins. Two heavy chain (KHC) and two light chain (KLC) subunits move cargoes unidirectionally in the anterograde direction while DYNLT3 is the major motor protein driving retrograde transport. An accumulation or aggregation of α-syn correlates with disturbances in transport along axons both in vitro an and in vivo conditions [10, 41, 49]. To study the putative effect of D-PUFAs on the levels of axonal transport-related proteins, membranes were incubated in the presence of KLC-1 and DYNLT3 antibodies (Fig. 5f–h). Immunoblotting analysis displayed a significant decrease in the immunoreactivity of KLC-1, and a trend to lower DYNLT3 protein levels in the α-syn transduced hemisphere relative to the GFP transduced side of H-PUFAs fed rats. Treatment with D-PUFAs abolished the α-syn-mediated axonal transport protein imbalance.

### D-PUFAs attenuate α-syn-induced inflammatory response

**Fig. 6:**
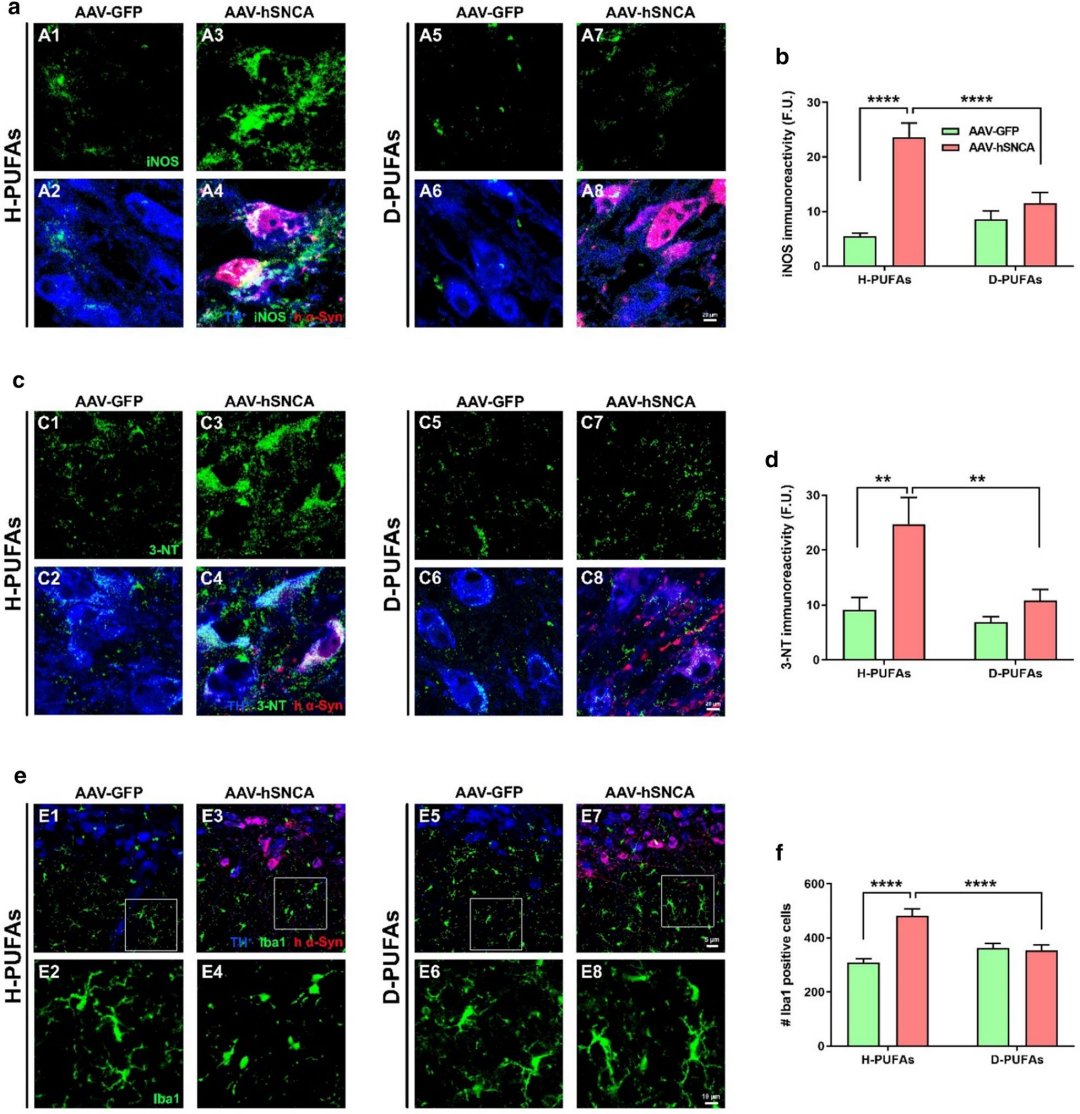
Sustained administration of D-PUFAs diminishes the levels of iNOS and 3-NT in SN DA neurons and mitigates α-syn-induced activation of microglia. **a** and **b** Representative confocal laser scanning micrographs acquired at 40 × illustrating the immunoreactivity of TH^+^ (blue), iNOS or 3-NT (green) and human α-syn (red) in the contralateral and ipsilateral rat SN. Bar graphs summarizing the fluorescence intensity of iNOS (**b**) and 3-NT (**d**). Scale bar: 20 μm. **e** Confocal image acquisition of midbrain sections immunostained using antibodies against TH^+^ (blue), Iba1 (green) and human α-syn (red) was performed at 20 ×. **f** Number of Iba1-positive cells. Insets represent a 4 × zoomed images. Scale bar: 5 μm low magnification; 10 μm for high magnification. An average of three midbrain sections per animal were evaluated and data from each tissue section were combined to determine means. Results are expressed as mean ± SEM of four different animals per group. ****p < 0.0001 and **p < 0.01. Two-way ANOVA followed by Tukey’s post-hoc multiple comparisons test

The enzyme activity and gene expression of iNOS play a pivotal role in orchestrating inflammation and are markedly increased in PD [49, 50]. Upregulation of iNOS leads to an excessive production of nitric oxide (NO^·^)—the primary source of reactive nitrogen species (RNS)—responsible for the activation of the downstream apoptotic signaling pathway. Free-radical mediated damage induces tyrosine nitration, a post-translational modification of proteins. Postmortem examinations of the brain tissue of human cases with PD exhibited a robust increase in 3-NT levels [23]. Therefore, we investigated the effects of D-PUFAs in the inflammatory process (Fig. 6). Midbrain sections were immunostained using an antibody against iNOS and were subjected to confocal microscopy analysis. In rats fed with a H-PUFAs diet, transduction with AAV-hA53T α-syn induced a five-fold increase in the fluorescence signal of iNOS while animals on a diet supplemented with D-PUFAs exhibited reduced iNOS levels (Fig. 6a, b). In addition, fluorescent micrographs depicted a significant increase in 3-NT immunoreactivity in the AAV-h*SNCA* hemisphere of rats treated with H-PUFAs, which was significantly decreased following dietary supplementation with D-PUFAs (Fig. 6c, d). To further assess the role of D-PUFAs in neuroinflammation, SN sections were labeled with an Iba1 antibody, a marker of activated microglia. Overexpression of α-syn elicited reactive phenotypes in microglia, as evidenced by a large number of Iba1-expressing cells (Fig. 6e, f). Microglial stimulation was mitigated upon treatment with D-PUFAs.

### D-PUFAs-mediated neuroprotective effect is independent of sirtuins

Sirtuins are a group of proteins that act mainly as nicotinamide adenine dinucleotide (NAD^+^)-dependent deacetylases that dynamically regulate transcription, metabolism, and cellular stress response. Sirtuins 1 and 2 are primary cytosolic while sirtuins 3, 4, and 5 are located in the mitochondria. There is increasing evidence that sirtuins play a key role in neurodegenerative diseases, such as PD [35]. We investigated the effects of D-PUFAs on regulating the levels of sirtuins (Additional file 1: Figure S4). No changes in the sirtuin gene expression were observed in the hemispheres of AAV-h*SNCA* rats treated with either H-PUFAs or D-PUFAs.

## Discussion

α-Syn misfolding and aggregation, lasting oxidative damage and mitochondrial alterations are considered to be primary pathogenic mechanisms implicated in DA-mediated neurodegeneration in PD. Overexpression of α-syn replicates many of the cardinal pathological features of human PD, including oxidative injury and mitochondrial dysfunction. Because DA neurons are significantly susceptible to LPO, we surmised that blocking the oxidation of lipids by administration of D-PUFAs might exert neuroprotective effects in PD. We have provided compelling evidence that dietary supplementation with D-PUFAs improves behavioral abnormalities and attenuates nigrostriatal DA neurodegeneration in a progressive model of PD.

Our findings show that AAV-driven overexpression of human mutated A53T α-syn into the rat SN over a period of 3 months causes a significant increase in the levels of endogenous α-syn but. However, high molecular weight species of α-syn or post-translational modifications, such as phosphorylation at Serine 129, were not detected. These contradictory findings may be partially explained by the differences in the virus concentration, infection time duration, delivery brain region and type of animal used. Moreover, transient transfections or viral-mediated overexpression of α-syn does not generate inclusions in primary neuronal cultures or neurons plated from transgenic mice expressing either wild-type or mutant α-syn. The bulk of the studies showing increased p-S129 levels were performed in genetic models but not using AAV-driven overexpression of α-syn. Moreover, it has been reported that low titers of rAAV-hSNCA cause a moderate and slow increase in the number of SN pS129 α-syn-positive cells [39]. Clinical evidence supports the notion that α-syn phosphorylation may occur after the formation of LBs and suggests that p-S129 accumulation could represent a late event in the disease progression [4, 56]. Our rats received a stereotactic injection of AAV-α-syn at 4 months of age and sacrificed at 7 months of age (an equivalent of an early onset PD). Other studies have shown that intrastriatal inoculation of preformed α-syn fibrils only increased the immunoreactivity of phosphorylated α-syn after 90 days post-injection [33].

α-Syn deposition into insoluble aggregates led to marked decrease of presynaptic and postsynaptic-related proteins in the cortex of an early-onset cohort of patients with Lewy body dementia (LBD) [30]. mRNA expression levels of a number of synaptic proteins are significantly downregulated in the SN of PD patients classified as Braak α-syn stages ranging from 0 to 6 [16]. An elevated content of α-syn at the synapses is associated with a redistribution and accumulation of SNAP-25, VAMP-2 and syntaxin-1 in the striatum of PD patients and α-syn transgenic mice [22]. Synthetic α-syn fibrils caused a significant decrease in the immunoreactivity of CSPα, synaptophysin, SNAP-25 and VAMP2 in primary ventral midbrain neuronal cultures [49]. Our results show that targeted overexpression of α-syn in the rat SN diminishes the content of CSPα and SNAP-25 synaptic proteins.

Excessive formation of ROS/RNS is the leading cause of oxidative stress-induced DA neuronal damage. 4-HNE and 4-HHE can readily react with thiols and amino groups of cysteine, lysine or histidine residues of proteins to generate stable Michael adducts. Abnormal concentration of 4-HNE and 4-HHE induces redox imbalance, mitochondrial respiratory failure, DNA fragmentation and apoptosis. 4-HNE adducts were present in the brainstem and neocortical LBs of subjects with diffuse LBD and PD [9]. An increased concentration of 4-HNE was detected in the SN DA neurons of individuals with PD [54]. Animal models of PD replicate the increased levels of 4-HNE seen in humans [48, 50]. Furthermore, excessive production of 4-HHE was found in the hippocampus of early-onset AD patients [7]. Incubation with 4-HHE resulted in protein adduction, increased ROS production and glutathione pool depletion in rat primary cortical neuronal cultures [32]. Our findings are in agreement with another study, in which intraperitoneal injection of the mitochondrial division inhibitor-1 (mdivi-1), an inhibitor of Drp1, rescued synaptic dysfunction and decreased the levels of 4-HNE in the brains of AAV-hA53T α-syn rats [6].

LPO products are chemically unstable and undergo rapid metabolism while different products derived of oxidative injury, such as isoprostanes and prostaglandins, show a stable metabolic profile that make them a more suitable markers of oxidative stress. PGs are lipid mediators generated from arachidonic acid in a reaction catalyzed by cyclooxygenase. Chronic microglial activation or astrocytic gliosis results in a steady stream-like release of high levels of prostaglandins, including PGE2α and PGF2α [25]. Although no changes have been observed in the CSF, upregulated levels of PGE2α were detected in the SN of individuals with PD [34]. IsoP are prostaglandin-like compounds produced by non-enzymatic peroxidation of arachidonic acid independent of the cyclooxygenase pathway and are regarded as the gold standard for detection of excessive endogenous LPO. Elevated levels of IsoP have been described in the anterior cingulate cortex of individuals afflicted with PD [1]. We found that stereotaxic injection of α-syn elicits LPO and oxidative injury, as evidenced by both increased F2-IsoPs and PGF2α, which were mitigated by D-PUFAs.

Oxidative damage results in mitochondrial dysfunction by disrupting the electron transport chain with subsequent electron leakage from donor redox centers to molecular oxygen. A selective decline in complex I activity was reported in the SN of PD patients [44]. Complex I is composed of several nuclear and mitochondrial DNA-encoded subunits; polymorphisms in mtDNA-encoded complex I constitute risk susceptibility factors for PD. Partial complex I deficiency was associated with impaired mtDNA transcription and replication and reduced mtDNA copy number in the SN of idiopathic PD patients [24]. DA neurons accumulate higher amounts of somatic mtDNA deletions and exhibit lower numbers of wild-type mtDNA in patients with PD as compared to healthy controls [17]. α-Syn contains a mitochondrial targeting sequence in its N-terminal region and regulates mitochondrial function by modulating respiratory chain complexes. Time-dependent accumulation of α-syn impaired complex I activity and increase the susceptibility to oxidative damage in DA neurons in vitro and in PD brains [13]. Our findings showed a significant α-syn-induced accumulation of ROS/RNS.

Mitochondrial dynamics and axonal transport are tightly interconnected and play a prominent role in preserving mitochondrial morphology and quality control. Mitochondrial fusion, governed by mitofusins and Opa1, facilitates mitochondrial content exchange, accumulation of mtDNA mutations and excessive ROS levels. The GTPases Drp1 and Fis1 are key mediators of mitochondrial fission, which results in fragmented mitochondria, oxidative damage and mitophagy. Axonal transport, a cellular mechanism controlled by the motor proteins kinesin and dynein, is responsible for the bidirectional movement of neurotransmitters, lipids, proteins and organelles. Perturbations in mitochondrial dynamics and disrupted motor-cargo interactions have been described in neurodegenerative diseases, including PD. Ablation of Mfn2 in mice caused behavioral deficits, nigrostriatal DA neurodegeneration, mitochondrial fragmentation and reduced movement along neuronal processes [40].

Evidence indicates that α-syn misfolding is linked to defective transport along axons, which in turn affects mitochondrial dynamics, leading to increased fission. α-syn accumulation elicited mitochondrial fragmentation in an age-dependent fashion and reduced the content of elongated mitochondria upon overexpression of Mfn1, Mfn2 and Opa1 fusion-promoting proteins [27]. It has been suggested that α-syn directly interacts with mitochondrial membranes to induce mitochondrial fragmentation through a Drp1-independent mechanism [37]. Expression of A53T α-syn in human neural stem cells increased the mitochondrial fragmentation phenotype although it did not alter Mfn2 and Drp1 protein levels [41]. Alterations in the content of both axonal transport- and mitochondrial dynamic-associated proteins were observed in primary ventral midbrain neuronal cultures incubated with synthetic α-syn fibrils [49]. Our findings showed that A53T α-syn overexpression results in impaired mitochondrial function and axonal transport.

Chronic release of proinflammatory cytokines by astrocytes and activated microglia contributes to the degeneration of SN DA neurons. Postmortem neuropathological examination showed the presence of reactive microglia around the SN α-syn aggregates in a cohort of PD patients [18]. α-syn overexpression in the SN of mice significantly increased the number of CD68-positive cells and triggered a neuroinflammatory response [51]. Proteome and transcriptome analyses indicated that nitration and deposition of α-syn into insoluble aggregates promotes microglial stimulation and its derived products, including cytokines and chemokines, in the SN of individuals with PD [43]. An elevated microglial response occurred in parallel with an induction of a proinflammatory molecular cascade in the SN of young transgenic mice overexpressing α-syn [47]. Transcriptional upregulation of iNOS by inflammatory stimuli in both neuronal and glial cells generates sustained large amounts of NO^•^ and RNS. Autopsied brains from subjects with PD and parkinsonized rodents contain increased levels of iNOS and proinflammatory cytokines [26, 48, 50]. Data from postmortem studies exhibited an increase in protein nitration in LB-bearing neurons in PD patients [23]. Upregulated content of 3-NT, a marker of protein modification by RNS, has been described in animal models of PD [48, 50]. Treatment with α-syn fibrils upregulated the levels of some specific pro-inflammatory mediators and the immunoreactivity of iNOS and 3-NT, suggesting that inflammation-derived oxidative stress and toxicity are involved in DA cell loss [49]. Our data showed that AAV-A53T α-syn induces neuroinflammation.

Due to its high concentration of PUFAs, LPO is a primary outcome of free radical-induced brain injury. Increased LPO of PUFAs was reported in the SN of individuals afflicted with PD [14]. PUFAs can interact with the N-terminal region of α-syn thereby promoting its oligomerization in rat mesencephalic neurons, transgenic mice overexpressing α-syn, and PD and DLB postmortem samples [45]. Long-term treatment with a docosahexaenoic acid (DHA)-enriched diet did not reduce α-syn levels but improved lifespan and decreased the amount of some synaptic-associated proteins in mice overexpressing human α-syn under the Thy-1 promoter [11]. Exposure to DHA altered brain lipid composition thereby diminishing the content of total PUFAs and oxidized proteins, although did not ameliorated PD-like phenotype in transgenic mice harboring human α-syn with the A53T mutation [36]. A53T α-syn transgenic mice fed with high DHA diet showed an accumulation of α-syn, an increase in the synaptophysin immunoreactive signal, and an activation of astrocytic cells via the activation of retinoic X receptor activation and the peroxisome proliferator-activated receptor γ2 [53]. Although preclinical data using experimental models of PD provided evidence of a beneficial effect of PUFAs in neurodegenerative diseases, clinical trials have failed to show a therapeutic benefit. Clinical trials of mild-to-moderate AD individuals found no evidence that ω-3 PUFAs improved cognition, function or dementia severity [8]. Double-bind, placebo-controlled studies demonstrated that ω-3 PUFAs dietary administration reduced symptoms of depression but did not ameliorate either the unified PD UPDRS rating or the Hoehn and Yahr scales [12].

In this work, we utilized stabilized PUFAs following substitution of deuterium for hydrogen at oxidation-prone bis-allylic sites, a process that results in strengthening of the C–H bonds (the first bonds dissociated during LPO). We showed that dietary intake of isotopically modified PUFAs decreases LPO and the associated H_2_O_2_-induced toxicity in a rat model of PD. Treatment with D-PUFAs restored the mitochondrial membrane potential in PLA2G6 (phospholipase A2 group VI) mutant human fibroblasts, and partially rescues the locomotor abnormalities in iPLA2-VIA null flies [28]. Cognitive performance and the cortical and hippocampal levels of F2-IsoPs and PGF2α were normalized following D-PUFAs supplementation in aldehyde dehydrogenase 2 deficient mice [19]. Supplementation with isotope‐reinforced PUFAs led to an increase in mitochondrial HSP60 immunoreactivity and protected DA content and fiber density in the striatum and DA neurons in the SN against the deleterious effects of MPTP [46]. Exogenous administration of D-PUFAs into primary neuron-glia co-cultures from rat cortex exhibited efficacy against oligomeric α-syn-mediated cell death and LPO [2].

## Conclusions

We report that viral vector-driven overexpression of human mutant A53T α-syn in the rat SN causes PD-like motor abnormalities, DA neurodegeneration, synaptic pathology, oxidative damage, mitochondrial dysfunction, perturbations on mitochondrial trafficking along axons and neuroinflammation. Our study provides proof of-concept data that isotopic reinforcement of essential PUFAs have a potent neuroprotective effect against α-syn-induced PD-like pathology. Our findings suggest that mitigation of oxidative damage mediated by LPO and preservation of mitochondrial integrity are mechanisms involved in the protective response within the nigrostriatal DA pathway. The protective action of D-PUFAs was independent of sirtuins. D-PUFAs have a safe and tolerable profile, with no off-target activities. Indeed, in a phase I/II double-blind randomized controlled trial, Friedreich's ataxia patients that received oral administration of RT001 (a deuterated ethyl linoleate) for 28 days show a significant peak workload improvement, with no adverse effects and both excellent safety and tolerability [55]. Our results support the advancement of dietary supplementation with D-PUFAs into clinical trials to evaluate their efficacy as a neuroprotective agent for PD.

## Supplementary information

**Additional file 1: Figure S1**. Protective effect of D-PUFAs on LPO.** Figure S2**. AAV-mediated overexpression in the rat brain.** Figure S3**. Long-term dietary supplementation with D-PUFAs has no effect on mitochondrial function in the striatum of AAV-A53T α-syn injected rats.** Figure S4**. D-PUFAs do not modulate sirtuin mRNA expression levels.** Table S1. Rat-based diets**. The formulation and nutrient content of the H-PUFAs and DPUFAs diets.** Table S2. Primary antibodies**. Antibodies used for immunohistochemical staining and Western blotting.** Table S3. Secondary antibodies**. Antibodies used for immunohistochemical staining and Western blotting.

## Abbreviations

AAV: Adeno-associated viral vector
CSP-α: Cysteine string protein alpha
D: Deuterated
DA: Dopamine
DHA: Docosahexaenoic acid
Drp1: Dynamin-related protein 1
GFP: Green fluorescence protein
H: Non-deuterated
4-HHE: 4-Hydroxy-2-hexenal
4-HNE: 4-Hydroxy-2-nonenal
Iba1: Ionized calcium-binding adapter molecule 1
iNOS: Inducible nitric oxide synthase
iPSCs: Induced pluripotent stem cells
IsoP: Isoprostanes
KHC: Kinase heavy chain
KLC: Kinase light chain
LBs: Lewy bodies
LBD: Lewy body dementia
LPO: Lipid peroxidation
MDA: Malondialdehyde
Mfn2: Mitochondrial mitofusin 2
MS: Mass spectrometry
NAD^+^: Nicotinamide adenine dinucleotide
NO^•^: Nitric oxide
3-NT: 3-Nitrotyrosine
Opa1: Optic atrophy 1
PD: Parkinson’s disease
PFB: Pentafluorobenzyl ester
PUFAs: Polyunsaturated fatty acids
PG: Prostaglandins
ω-3: Omega 3
ω-6: Omega 6
RNS: Reactive nitrogen species
ROS: Reactive oxygen species
SN: Substantia nigra
SNAP25: Synaptosomal-associated protein 25
α-Syn: α-Synuclein
TMS: Trimethylsilyl ether
TH: Tyrosine hydroxylase
