# Supplementary material for: Isotope-reinforced polyunsaturated fatty acids improve Parkinson’s disease-like phenotype in rats overexpressing α-synuclein
Source: Acta Neuropathol Commun. 2020 Dec 11;8:220. doi: 10.1186/s40478-020-01090-6 (PMC7731572; doi:10.1186/s40478-020-01090-6)
Supplement: Supplementary file 1 — Additional file 1: Figure S1. Protective effect of D-PUFAs on LPO. Figure S2. AAV-mediated overexpression in the rat brain. Figure S3. Long-term dietary supplementation with D-PUFAs has no effect on mitochondrial function in the striatum of AAV-A53T α-syn injected rats. Figure S4. D-PUFAs do not modulate sirtuin mRNA expression levels. Table S1. Rat-based diets. The formulation and nutrient content of the H-PUFAs and DPUFAs diets. Table S2. Primary antibodies. Antibodies used for immunohistochemical staining and Western blotting. Table S3. Secondary antibodies. Antibodies used for immunohistochemical staining and Western blotting. [file 40478_2020_1090_MOESM1_ESM.docx]

**Isotope-Reinforced Polyunsaturated Fatty Acids Improve Parkinson’s Disease-Like Phenotype in Rats Overexpressing α-Synuclein**

**Authors and affiliations**

M. Flint Beal**^1,2^**, Jayandra Chiluwal**^1,2^**, Noel Y. Calingasan**^1,2^**, Ginger L. Milne**^3^**, Mikhail S. Shchepinov**^4^**, Victor Tapias**^1,2*^**

^1^Feil Family Brain and Mind Research Institute, Weill Cornell Medicine, New York, NY, 10065, USA; ^2^Deparment of Neurology and Neuroscience, Weill Cornell Medicine, New York, NY, 10065, USA; ^3^Division of Clinical Pharmacology, Vanderbilt University Medical Center, Nashville, TN, 37232; ^4^Retrotope, Inc., Los Altos, CA, 94022

**Running title**

D-PUFAs are neuroprotective in PD

**^*^Corresponding author**

Victor Tapias, PhD

Assistant Professor of Neuroscience

Feil Family Brain and Mind Research Institute

Weill Cornell Medicine

1300 York Ave, A-501

New York, NY 10065

Tel: 001-212-746-5341

Fax: 001-212-746-8276

Email: vit2013@med.cornell.edu

ORCID: [0000-0002-1783-7320](http://orcid.org/0000-0002-1783-7320)

**Supplementary Material**

**Figure S1.** **Protective effect of D-PUFAs on LPO.** (**A**) D-PUFAs prevent the rate-limiting step of ROS-driven abstraction of bis-allylic sites. (**B**) A lipid bilayer that incorporates D-PUFAs (ω-6 PUFAs D2-linoleic acid) is resistant to LPO. (**C**) Hydrogen abstraction at bis-allylic sites generates resonance-stabilized free radicals, which react with molecular oxygen to form lipid peroxyl radicals (LOO^•^) (**D**). LOO^•^ abstract a hydrogen from another PUFA to generate hydroperoxides (LOOH) (**E**). Linoleic acid (18:2, ω-6) or 11,11-D2-linoleic acid ethyl (**F**) are enzymatically converted into arachidonic acid and 13,13-D2-arachidonic acid (20:4, ω-6), α-linolenic acid (18:3, ω-3) or 11,11,14,14-D4-linolenic acid ethyl esters (**G**) which are enzymatically transformed into higher ω-3 PUFAs. (**H**) Lipid peroxides can decompose through multiple pathways into numerous species, including 4-HNE (**a**), 4-HHE (**b**) and MDA, which predominantly exists in one tautomeric form (**c**), acrylic aldehyde (**d**), oxalic aldehyde (**e**) and methylglyoxal (**f**). Other classes of products include arachidonic acid-derived isoprostanes iPF2α-IV or 8-IsoP (**g**) and PGF2α (**h**).

**Figure S2.** **AAV-mediated overexpression in the rat brain.** (**A**) AAV5.2 vector encoding a GFP reporter gene showed extensive transduction of SN DA neurons. Scale bar: 20 μm (**B**) Viral transduction efficiency in the striatum. Scale bar: 100 μm.

**Figure S3.** **Long-term dietary supplementation with D-PUFAs has no effect on mitochondrial function in the striatum of AAV-A53T α-syn injected rats.** (**A**) Immunoblotting for OXPHOS complexes. No changes were observed in the immunoreactivity of the respiratory chain complexes after treatment with D-PUFAs. (**B**) Histogram showing the levels of the target proteins. (**C**) Quantification of the mtDNA copy number using RT-qPCR analysis. Data are expressed as mean ± SEM of three animals per group. Two-way ANOVA followed by Tukey’s post-hoc multiple comparisons test.

**Figure S4.** **D-PUFAs do not modulate sirtuin mRNA expression levels.** Gene expression of SIRT1, SIRT3, SIRT4 and SIRT5 in the SN (**A**) and striatum (**B**).

**Table S1. Rat-based diets.** The formulation and nutrient content of the H-PUFAs and D-PUFAs diets.

**Table S2. Primary antibodies.** Antibodies used for immunohistochemical staining and Western blotting.

**Table S3. Secondary antibodies.** Antibodies used for immunohistochemical staining and Western blotting.

**Figure S1.**


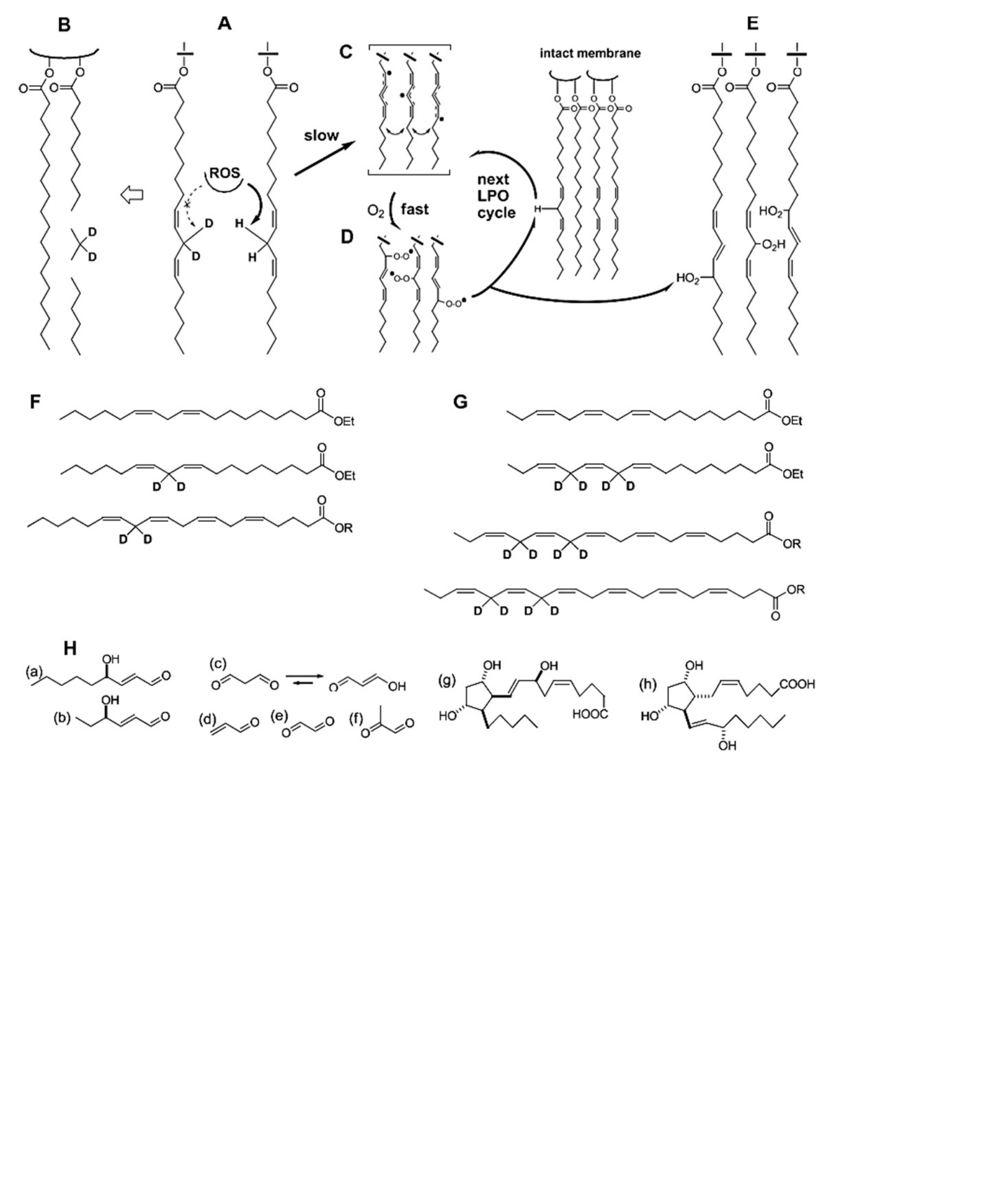


**Figure S2.**


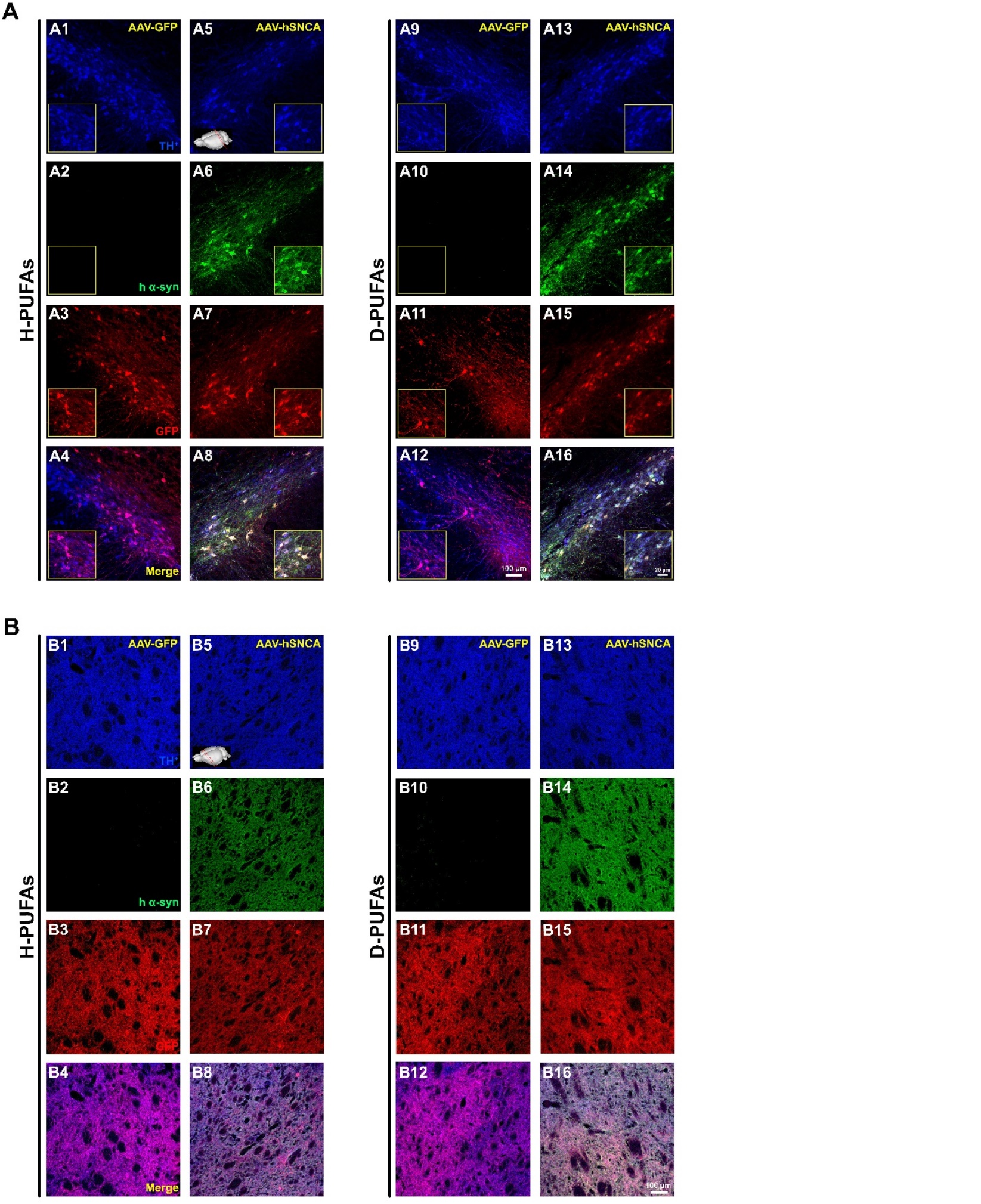


**Figure S3.**


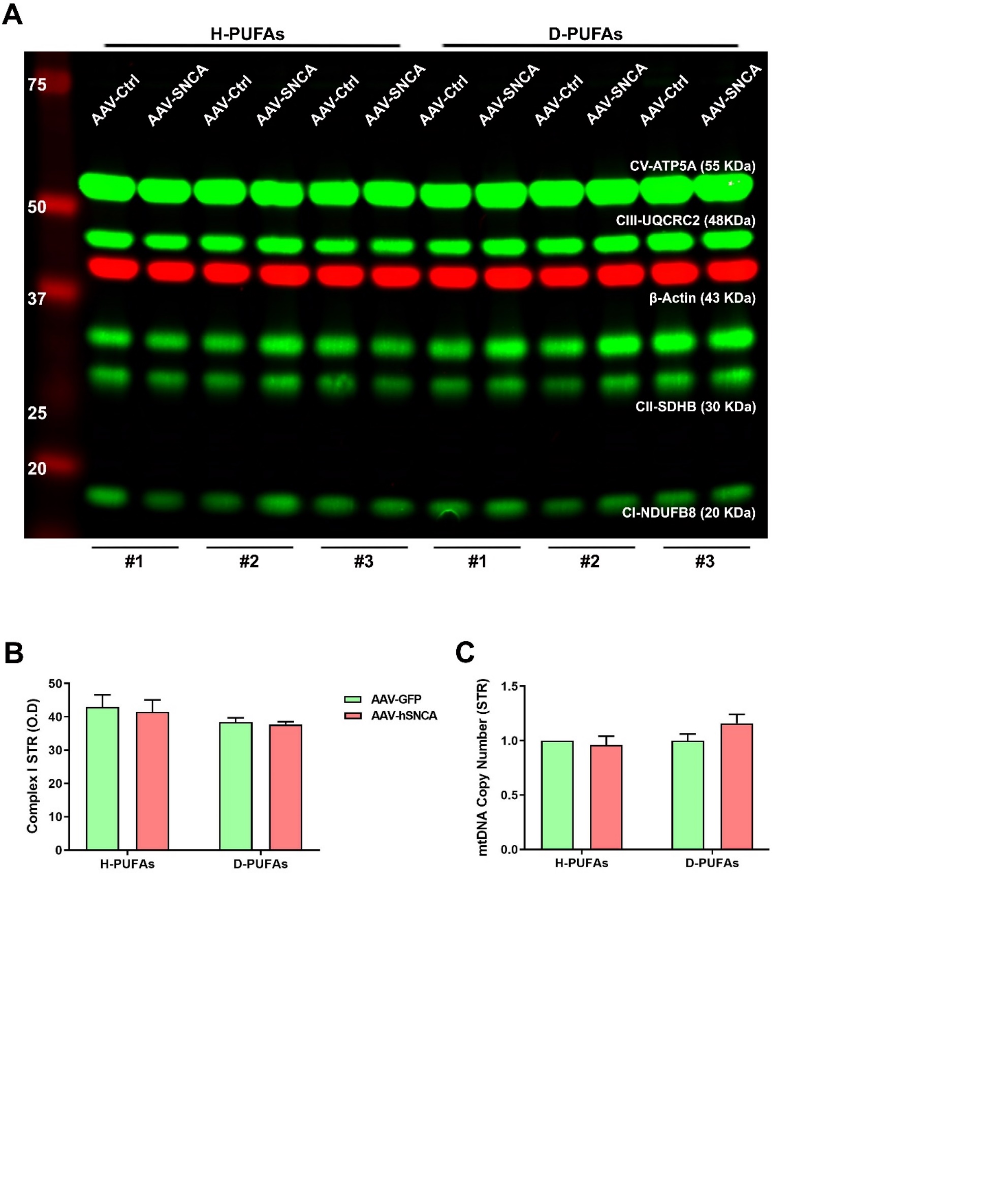


**Figure S4.**


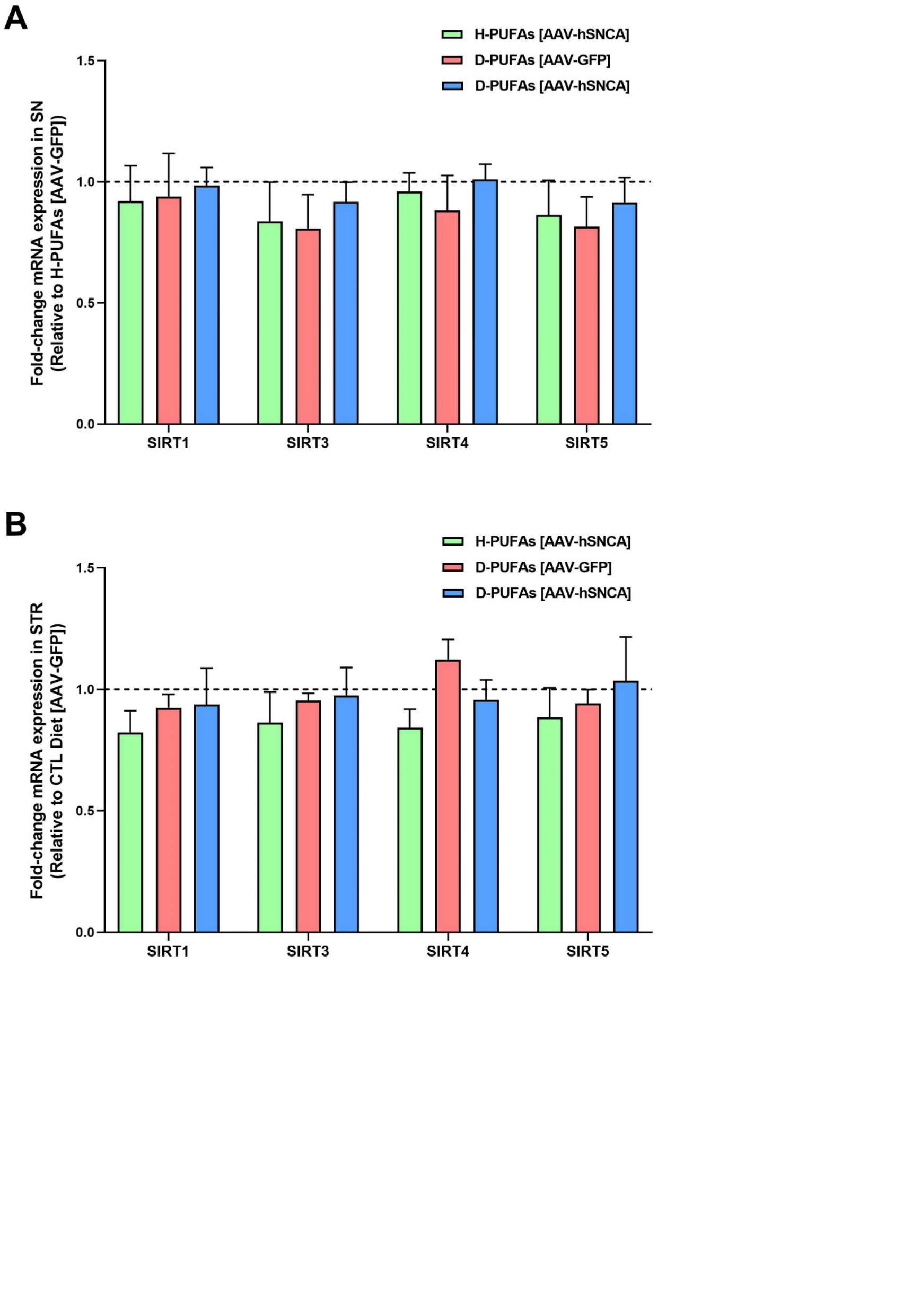


**Table S1. AIN-93M Rodent-based diets**

|  | **H-PUFAs diet** | | **D-PUFAs diet (1:1)** | |
| --- | --- | --- | --- | --- |
| **Nutrients** | **g (%)** | **Kcal (%)** | **g (%)** | **Kcal (%)** |
| **Carbohydrates** | **67** | **65** | **67** | **65** |
| **Proteins** | **14** | **13** | **14** | **13** |
| **Fat** | **10** | **22** | **10** | **22** |
| **Total** |  | **100** |  | **100** |
| **Kcal/g** | **4.2** |  | **4.2** |  |
|  |  |  |  |  |
| **Ingredients** | **g** | **Kcal** | **g** | **Kcal** |
| **D-linoleic acid, ethyl ester** | **0** | **0** | **8** | **72** |
| **D-linolenic acid, ethyl ester** | **0** | **0** | **0** | **0** |
| **H-linoleic acid, ethyl ester** | **8** | **72** | **0** | **0** |
| **H-linolenic acid, ethyl ester** | **3** | **27** | **3** | **27** |
| **Oleate, ethyl** | **25** | **225** | **25** | **225** |
| **Coconut oil, 101 (hydrogenated)** | **65** | **585** | **65** | **585** |
| **Casein** | **140** | **560** | **140** | **560** |
| **L-cystine** | **1.8** | **7.2** | **1.8** | **7.2** |
| **Corn starch** | **447** | **1788** | **447** | **1788** |
| **Maltodextrin 10** | **125** | **500** | **125** | **500** |
| **Sucrose** | **100** | **400** | **100** | **400** |
| **Cellulose, BW200** | **50** | **0** | **50** | **0** |
| **t-butylhydroquinone** | **0.008** | **0** | **0.008** | **0** |
| **Mineral Mix S10022M** | **35** | **0** | **35** | **0** |
| **Vitamin Mix V10037** | **10** | **40** | **10** | **40** |
| **Choline bitartrate** | **2.5** | **0** | **2.5** | **0** |
| **FD&C Blue Dye #1** | **0** | **0** | **0.05** | **0** |
| **Total** | **1012.308** | **4204** | **1012.308** | **4204** |

| **Antibody** | **Host** | **IHC dilution** | **WB dilution** | **Source** |
| --- | --- | --- | --- | --- |
| **TH** | Mouse monoclonal, C-LNC1  Rabbit polyclonal  Sheep polyclonal | 1:2000  1:3000  1:2000 |  | MAB318, EMD Millipore  AB152, EMD Millipore  AB1542, EMD Millipore |
| **GFP**  **Human α-syn**  **α-syn**  **α-syn pSer129** | Rabbit polyclonal  Mouse monoclonal, C-Syn211  Rabbit monoclonal  Mouse monoclonal  Rabbit polyclonal | 1:5000  1:4000  1:2000 | 1:1000  1:1000 | AB3080P, EMD Millipore  36-008, EMD Millipore  Ab138501, Abcam  610787, BD Biosci  PPS091, R&D Systems |
| **CSP-α**  **SNAP25**  **4-HNE**  **4-HHE**  **OXPHOS**  **Mfn2**  **Opa1**  **Drp1**  **KLC1** | Rabbit polyclonal  Mouse monoclonal, C-SP14  Mouse monoclonal  Mouse monoclonal  Mouse monoclonal  Rabbit polyclonal, C-D2D10  Goat polyclonal, C-15  Mouse monoclonal, C-5  Rabbit polyclonal | 1:500  1:1000  1:2000  1:3000  1:2000 | 1:1000  1:1000  1:1000  1:1000 | AB1576, EMD Millipore  MAB331, EMD Millipore  24327, Percipio Biosci.  NBP2-59352, Novus Biol.  Ab110413, Abcam  9482S, Cell Signaling  sc-30573, Santa Cruz  sc-271583, Santa Cruz  AP8637c, Abcepta |
| **DYNLT3** | Mouse monoclonal |  | 1:1000 | MAB1077, EMD Millipore |
| **iNOS**  **3-NT** | Mouse monoclonal  Rabbit polyclonal | 1:5000  1:500 |  | ab49999, Abcam  06-284, EMD Millipore |
| **Iba1**  **β-Actin** | Rabbit polyclonal  Mouse monoclonal, C-4  Rabbit polyclonal | 1:20000 | 1:25000  1:25000 | 019-19741, Wako  MAB1501, EMD Millipore  Ab8227, Abcam |

**Table S2.** **Primary antibodies used for immunohistochemical staining and Western blotting**

**Table S3.** S**econdary antibodies used for immunohistochemical staining and Western blotting**

| **Antibody** | **Host** | **IHC dilution** | **WB dilution** | **Source** |
| --- | --- | --- | --- | --- |
| **Alexa Fluor 488** | Donkey anti-Mouse  Donkey anti-Rabbit  Donkey anti-Sheep | 1:500  1:500  1:500 |  | A-21202, Thermo Fisher  A-21206, Thermo Fisher  A-11015, Thermo Fisher |
| **Cy3 (543)** | Donkey anti-Mouse  Donkey anti-Rabbit  Donkey anti-Goat | 1:500  1:500  1:500 |  | 715-165-151, Jackson ImmunoRes  711-165-152, Jackson ImmunoRes  705-165-147, Jackson ImmunoRes |
| **Alexa Fluor 647** | Donkey anti-Mouse  Donkey anti-Rabbit | 1:500  1:500 |  | A-31571, Thermo Fisher  A-31573, Thermo Fisher |
| **Biotinylated** | Goat anti-Rabbit | 1:200 |  | BA-1000, Vector Labs |
| **IRDye 680RD** | Donkey anti-Mouse  Donkey anti-Rabbit |  | 1:10000  1:10000 | 926-68072, LI-COR  926-68073, LI-COR |
| **IRDye 800CW** | Donkey anti-Mouse  Donkey anti-Rabbit  Donkey anti-Sheep  Donkey anti-Goat |  | 1:10000  1:10000  1:10000  1:10000 | 926-32212, LI-COR  926-32213, LI-COR  613-745-168, Rockland  926-32214, LI-COR |
